# The utility of high-frequency 18 MHz ultrasonography for preoperative evaluation of acral melanoma thickness in Chinese patients

**DOI:** 10.3389/fonc.2023.1185389

**Published:** 2023-10-05

**Authors:** Nianzhou Yu, Kai Huang, Yixin Li, Zixi Jiang, Siliang Liu, Yuancheng Liu, Xiaowan Liu, Zeyu Chen, Renliang He, Tianhong Wei

**Affiliations:** ^1^ Department of Dermatology, Hunan Engineering Research Center of Skin Health and Disease, Hunan Key Laboratory of Skin Cancer and Psoriasis, Xiangya Hospital, Central South University, Changsha, Hunan, China; ^2^ National Clinical Research Center for Geriatric Disorders, Xiangya Hospital, Central South University, Changsha, Hunan, China; ^3^ State Key Laboratory of High Performance Complex Manufacturing, College of Mechanical and Electrical Engineering, Central South University, Changsha, China; ^4^ Department of Dermatologic Surgery, Dermatology Hospital of Southern Medical University, Guangzhou, Guangdong, China; ^5^ Department of Ultrasound, Xiangya Hospital, Central South University, Changsha, Hunan, China

**Keywords:** acral melanoma, high-frequency, ultrasonography, thickness, preoperative

## Abstract

**Background:**

Despite the increasing use of preoperative ultrasound evaluation for melanoma, there is limited research on the use of this technique for Acral Melanoma (AM).

**Methods:**

This retrospective study analyzed the electronic medical records of patients who underwent preoperative evaluation for cutaneous melanoma maximum thickness using an 18 MHz probe and histopathological examination between December 2017 and March 2021 at the Department of Dermatology in Xiangya Hospital, Central South University.

**Results:**

A total of 105 patients were included in the study. The mean tumor thickness was 3.9 mm (s.d., 2.3), with 63% of the specimens showing ulceration and 44 patients showing lymph node metastasis. The results showed a good correlation between the high-frequency ultrasonography (HFUS) and histopathological thickness measurements, with a Spearman’s correlation coefficient of 0.83 [(95% CI 0.73–0.90) (P < 0.001)]. The positive predictive value (PPV) of sonography in identifying tumor thickness was also found to be high.

**Conclusion:**

Our study suggests that high-frequency 18 MHz ultrasonography is an effective tool for the preoperative evaluation of AM thickness. The HFUS measurements correlated well with the histopathological thickness measurements, making it a valuable and reliable method for clinicians to assess the thickness of melanoma lesions preoperatively.

## Introduction

Cutaneous melanoma (CM) is a type of skin cancer that originates from cells in the skin that produce pigments called melanocytes (1, 2). CM can occur on any part of the skin, and it develops on the palms of the hands, soles of the feet, or under the nails and is called Acral Melanoma (AM). It is the most common type of melanoma in people with darker skin, such as those of African, Asian, or Hispanic descent (3). According to a study published recently, AM is the most common subtype of melanoma in Chinese individuals, accounting for approximately 52% of all melanoma cases in China (4). Breslow thickness is the most important prognostic factor with respect to overall survival for AM (5). The primary treatment of melanoma is surgical excision (SE), of which surgical margins are decided by Breslow thickness. The safety peripheral surgical margins according to the National Comprehensive Cancer Network (NCCN) Guidelines for CM are (2) 0.5-1.0 cm for *in situ* melanoma, 1 cm for melanoma ≤1 mm, 1-2 cm for melanoma 1-2 mm and 2 cm for melanoma >2 mm (6). Partial biopsy has a significantly lower diagnostic accuracy than complete excision biopsy, which is considered the gold standard for the diagnosis of skin tumors, including melanoma. The excision biopsy of AM can be challenging due to its relatively larger Breslow thickness and volume when compared to other melanoma subtypes (7, 8). Accurate determination of preoperative Breslow thickness in AM poses a significant clinical challenge. Current diagnostic tools and techniques have limited sensitivity and specificity, leading to potential underestimation or overestimation of Breslow thickness. This uncertainty can lead to suboptimal surgical planning and clinical decision-making. Therefore, there is an urgent need for improved diagnostic methods to better predict Breslow thickness in AM and improve clinical outcomes.

High-Frequency Ultrasonography (HFUS) is a noninvasive method that is mainly used to estimate tumor thickness in melanoma, to plan one-step excisions with appropriate margins, and to help determine the necessity of sentinel lymph node biopsy (9). The utilization of high-frequency ultrasound probes with frequencies exceeding 20 MHz has been demonstrated to be an appropriate modality for the preoperative assessment of melanoma (10, 11).

Despite the increasing use of preoperative ultrasound evaluation for melanoma, there is limited research on the use of this technique for AM. The purpose of this study was to evaluate the accuracy of melanoma thickness measurements obtained by 18 MHz probes in comparison to histopathological examination for this subtype of melanoma.

## Methods

### Study design

This retrospective study analyzed the electronic medical records of patients who underwent preoperative evaluation for cutaneous melanoma maximum thickness using an 18 MHz probe and histopathological examination between December 2017 and March 2021 at the Department of Ultrasound in Xiangya Hospital, Central South University. The study was conducted in compliance with the Declaration of Helsinki and approved by the Ethics Committee of the Department of Dermatology, Xiangya Hospital. Patients provided written informed consent. Ultrasound examination was performed using a 5-18 MHz linear transducer, and two sonologists with over five years of experience independently performed multiple measurements for the thickest area of suspiciousness and recorded the maximum thickness. Surgical excision followed the NCCN Guidelines for surgical margins, and wide surgical excision combined with a sentinel lymph node biopsy was used for melanoma stages T1b or higher. Histopathological examination was performed by an experienced dermatopathologist blinded to the measurements of HFUS.

### Ultrasound examination

**Figure 1:**
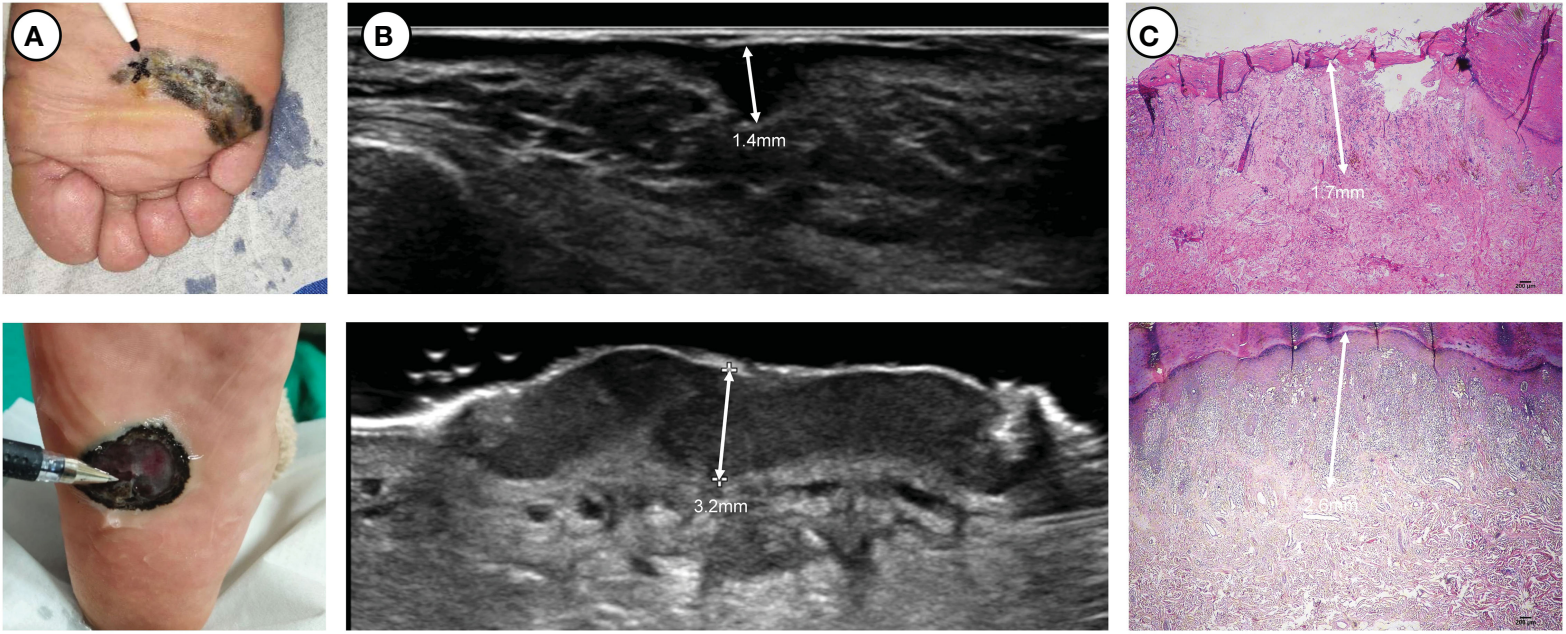
Two thick ulcerated nodular melanomas located on the plantar side: **(A)** clinical pictures, **(B)** ultrasound pictures and **(C)** pathological pictures. At the apex of the pen lies the location where ultrasonic measurements exhibit the maximum thickness.

A 5-18 MHz linear transducer (Philips, EPIQ5, 5-18 MHz, MSK Superficial) was used to examine the lesions. The width of the probe was 38 mm with a maximal depth of 60 mm. Standard sonographic gel was applied to separate the probe face from the skin surface. The probe was held manually and moved at least two directions over the skin to provide screening of the entire lesion. Two sonologists with over five years of experience independently scanned the lesion, performed multiple measurements for the thickest area of suspiciousness, and recorded the maximum thickness (**Figure 1**; **Figure S1**).

### Surgical excision

Histopathological examination was performed by an experienced dermatopathologist blinded to the measurements of HFUS. The surgical margins were determined based on the Breslow thickness from the pathology report of the incisional biopsy or excisional biopsy, and the safety peripheral surgical margins were in accordance with the NCCN Guidelines. Wide surgical excision combined with a sentinel lymph node biopsy was used for melanoma stages T1b or higher. This approach ensured thorough removal of the tumor while also assessing the potential spread to the nearby lymph nodes, as recommended by the NCCN Guidelines. The results of the included Breslow thickness were derived from the pathology reports of excisional biopsy as well as post-operative pathology reports following wide surgical excision (if incisional biopsy were performed).

### Statistical analysis

The data reported in the study are presented as frequencies, percentages, and means with standard deviations. To evaluate the correlation between tumor thickness measured by HFUS and histopathology, the researchers used Bland-Altman plots, Spearman’s correlation coefficient, and interrater agreement (kappa). Positive predictive values (PPVs) were also calculated to assess the accuracy of sonography in identifying tumor thickness. Statistical significance was set at P<0.05. All statistical analyses were performed using IBM SPSS 25.

## Result

A total of 115 patients were retrospectively reviewed for this study, although 10 patients were excluded due to incomplete ultrasound or pathological data. The remaining 105 patients (55 women, 50 men) with 109 lesions had a mean age of 61.8 (IQR 54.0-71.0) years. Most of the lesions (91.7%) were located in the upper and lower limbs, with a higher incidence in the plantar region (45.0%). The mean tumor thickness was 3.9 mm (s.d., 2.3). Additionally, 63% of the specimens had ulceration, and 44 patients had lymph node metastasis. The baseline characteristics are presented in **Table 1**.

**Table 1:** Baseline characteristics.

Characteristics	Variable		
**No. lesions**		109	
**No. patients**		105	
** Gender**	**M/F**	50 (47.6)	55(52.3)
** Age, years**	**Mean (IQR)**	61.8 (IQR 54.0-71.0)	
** Primary tumor** **location**	**Head and neck**	5 (4.6)	
	**Trunk**	4 (3.7)	
	**Upper limbs**	18 (16.5)	
	**Lower limbs**	82 (75.2)	
** Type of biopsy**	**Excision biopsy**	97 (89.0)	
	**Incision biopsy**	12 (11.0)	
** Breslow**	**Mean (SD)**	3.9 (2.3)	
** Clark’s level**			
	**II-III**	56 (51.4)	
	**IV-V**	53 (48.6)	
** Ulceration**	**Absent**	40 (36.7)	
	**Present**	69 (63.3)	
**Lymph node metastasis**	**No**	61 (58.1)	
	**Yes**	44 (41.9)	

Data are the mean (SD) or number (%).

IQR, interquartile range; SD, standard deviation.

### Comparison of measurements

**Figure 2:**
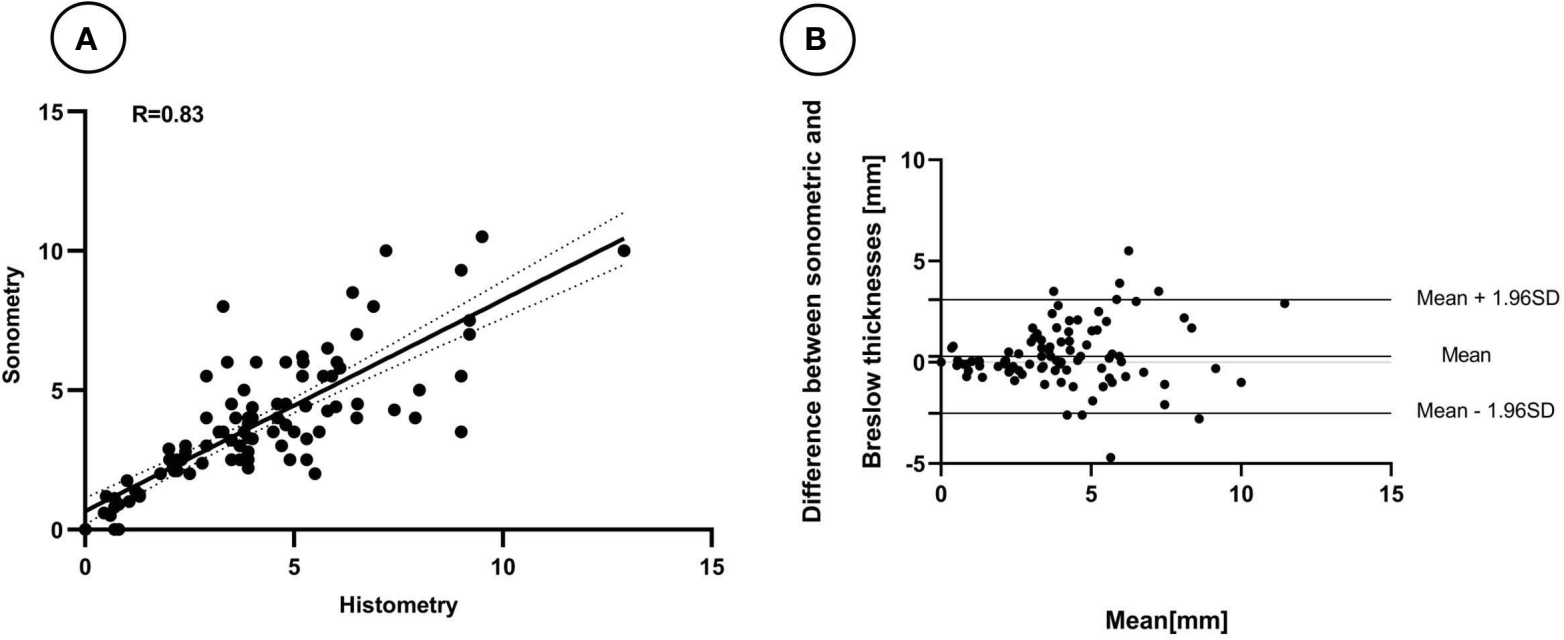
Linear relation **(A)** and Bland-Altman plots **(B)** between the thickness of melanomas measured by Breslow thicknesses and the 18 MHz probe ultrasound.

According to the NCCN Guidelines, the thickness of tumors can be categorized into five types (2). Our study found that 86 melanomas (78.9%) were classified in the same interval as the guidelines, and 99 melanomas (90.8%) would receive the correct surgical margin if guided by ultrasound measurement. However, the group of melanomas with thicknesses of 1-2 mm had a lower PPV, which was 50.0% (**Table 2**). We observed a better agreement between tumor thickness measurements obtained through ultrasound and histopathology using Bland-Altman plots and linear regression analysis (**Figure 2**). The Spearman’s coefficient of 0.83[(95% CI 0.73–0.90) (P < 0.001)] between ultrasound and histological tumor thickness was considered good.

**Table 2:** Concordance between sonographic thickness and histometric thickness measurements in 109 melanoma lesions.

Sonometry						
Histometry	in situ	≤1 mm	>1.00-2 mm	>2.00-4 mm	>4 mm	PPV*
in situ	3	2	0	0	0	60.0%
≤1 mm	0	7	1	0	0	87.5%
>1.00-2 mm	0	2	5	2	1	50.0%
>2.00-4 mm	0	0	2	36	9	76.5%
>4 mm	0	0	0	4	35	89.7%

*PPV, positive predictive value.

## Discussion

Probes with a frequency of 15 MHz or greater can provide clear delineation of skin layer morphology, including changes in epidermal thickness (12). Numerous prior investigations have established the efficacy of probes with frequencies of 20 MHz or higher in measuring tumor thickness (13). Additionally, even 12-15 MHz linear probes can reliably differentiate between melanomas thicker and thinner than 1 mm (14). For ultrasound with an 18 MHz frequency, the Spearman’s coefficient between ultrasound and histological tumor thickness was 0.83 (95% CI 0.73–0.90) (P < 0.001), which was not inferior to probes with higher frequencies used in other studies (12). Bland-Altman plots revealed a better agreement between both methods. Only 10 (9.2%) melanomas would have undergone incorrect surgical margin determination if guided by ultrasound measurement, demonstrating promising clinical prospects. Our study demonstrates that ultrasonic measurement of AM thickness using 15-18 MHz probes is highly effective. The unique location of AM may limit the depth of penetration of probes above 50 MHz, leading to inadequate evaluation. Conventional ultrasound devices utilizing probes with up to 18 MHz frequency have a significant advantage in evaluating intratumoral angiogenesis to identify melanomas with a high metastatic potential using color Doppler sonography (15). Unfortunately, such an inspection cannot be performed using devices with a probe frequency of 20 MHz or higher. In our previous research, we observed that the utilization of the 18MHz probe resulted in improved localization during incisional biopsies in patients with AM (16). This finding suggests that the use of the 18MHz probe can aid in accurately identifying the target area for biopsy, facilitating more precise and targeted sampling of the lesion. By enhancing the localization process, the 18MHz probe offers a potential benefit in optimizing diagnostic accuracy and ensuring appropriate tissue sampling in patients with AM. This information further supports the potential utility of the 18MHz probe in the management and evaluation of AM cases.

This study was conducted as a retrospective analysis of ultrasound data and pathological results, with the final determination of surgical margins still being made based on pathology. Our investigation revealed several limitations in the use of HFUS, including its potential to erroneously overestimate Breslow thickness and its lack of accuracy for thinner melanomas. Various factors, such as inflammatory infiltration in the lower portion, difficulties in holding the probe, or a thick stratum corneum, can affect the accuracy of ultrasound and are challenging to control. While small or normal-sized melanomas are not affected, larger melanomas in certain anatomical regions, such as the palm/sole, digit, face, and ear, may be impacted (17).

Our study has some limitations. First, overall, the accuracy of 18MHz in AM is not as high. The lowest PPV is only 50% for melanomas measuring 1-2mm in thickness. It is possible that a 20MHz probe may perform better in detecting thinner melanomas in acral regions (18). Second, the present study is the lack of a control group and a smaller sample size, rendering the conclusions less robust. Future efforts will involve collaboration with primary healthcare institutions to broaden the scope of the methodology and include a larger sample size for standard clinical investigations with long-term follow-up.

## Conclusion

The results of this study have shown that 18 MHz frequency sonography can be utilized as an uncomplicated and effective noninvasive technique for assessing acral melanoma prior to surgery. There exists a positive correlation between the thickness measurements obtained through 18 MHz ultrasound and histological analysis.

## Data availability statement

The raw data supporting the conclusions of this article will be made available by the authors, without undue reservation.

## Ethics statement

The studies involving humans were approved by the Ethics Committee of Xiangya Hospital of Central South University. The studies were conducted in accordance with the local legislation and institutional requirements. Written informed consent for participation in this study was provided by the participants’ legal guardians/next of kin.

## Author contributions

NY: data curation-equal, formal analysis-equal, visualization equal, writing-original draft-equal. KH: validation-equal, formal analysis-equal, writing-review & editing-equal. YXL: writing-review & editing-equal. ZJ: writing-review & editing equal, visualization-equal, resources-equal. SL: writing-review & editing-equal, visualization-equal, resources-equal. YCL: validation-equal, formal analysis-equal, writing-review & editing equal. XL: writing-review & editing-equal. ZC: formal analysis equal, visualization-equal, writing-review & editing-equal. RH: data curation-equal, formal analysis equal, visualization-equal, writing-review & editing-equal. TW: data curation-equal, formal analysis-equal, visualization-equal, writing review & editing-equal. All authors contributed to the article and approved the submitted version.

## References

[1] Swetter S Tsao H Bichakjian C Curiel-Lewandrowski C Elder D Gershenwald J. Guidelines of care for the management of primary cutaneous melanoma. J Am Acad Dermatol (2019) 80(1):208–50. doi: 10.1016/j.jaad.2018.08.055 30392755

[2] The NCCN melanoma: cutaneous clinical practice guidelines in oncology (version 2.2023 – march 10, 2023) (2023). National Comprehensive Cancer Network. Available at: http://www.nccn.org (Accessed May 10, 2023).

[3] Zhang Y Lan S Wu D. Advanced acral melanoma therapies: current status and future directions. Curr Treat options Oncol (2022) 23(10):1405–27. doi: 10.1007/s11864-022-01007-6 PMC9526689 36125617

[4] Wang Y Zhao Y Ma S. Racial differences in six major subtypes of melanoma: descriptive epidemiology. BMC cancer (2016) 16(1):691. doi: 10.1186/s12885-016-2747-6 27576582 PMC5004333

[5] Roka F Kittler H Cauzig P Hoeller C Hinterhuber G Wolff K. Sentinel node status in melanoma patients is not predictive for overall survival upon multivariate analysis. Br J cancer (2005) 92(4):662–7. doi: 10.1038/sj.bjc.6602391 PMC2361872 15700039

[6] Lees V Briggs J. Effect of initial biopsy procedure on prognosis in Stage 1 invasive cutaneous Malignant melanoma: review of 1086 patients. Br J surgery (1991) 78(9):1108–10. doi: 10.1002/bjs.1800780923 1933198

[7] Wei X Chen Y Yao H Wu D Li H Zhang R. Prognostic impact of Breslow thickness in acral melanoma: A retrospective analysis. J Am Acad Dermatol (2022) 87(6):1287–94. doi: 10.1016/j.jaad.2022.08.052 36075285

[8] Oh Y Choi S Cho M Nam K Shin S Chang J. Male sex and Breslow thickness are important risk factors for recurrence of localized melanoma in Korean populations. J Am Acad Dermatol (2020) 83(4):1071–9. doi: 10.1016/j.jaad.2019.09.029 31562946

[9] Adabi S Hosseinzadeh M Noei S Conforto S Daveluy S Clayton A. Universal in *vivo* Textural Model for Human Skin based on Optical Coherence Tomograms. Sci Rep (2017) 7(1):17912. doi: 10.1038/s41598-017-17398-8 29263332 PMC5738372

[10] Machet L Belot V Naouri M Boka M Mourtada Y Giraudeau B. Preoperative measurement of thickness of cutaneous melanoma using high-resolution 20 MHz ultrasound imaging: A monocenter prospective study and systematic review of the literature. Ultrasound Med Biol (2009) 35(9):1411–20. doi: 10.1016/j.ultrasmedbio.2009.03.018 19616369

[11] Serrone L Solivetti F Thorel M Eibenschutz L Donati P Catricalà C. High frequency ultrasound in the preoperative staging of primary melanoma: a statistical analysis. Melanoma Res (2002) 12(3):287–90. doi: 10.1097/00008390-200206000-00013 12140386

[12] Jasaitiene D Valiukeviciene S Linkeviciute G Raisutis R Jasiuniene E Kazys R. Principles of high-frequency ultrasonography for investigation of skin pathology. J Eur Acad Dermatol Venereol JEADV (2011) 25(4):375–82. doi: 10.1111/j.1468-3083.2010.03837.x 20849441

[13] Hinz T Ehler L Voth H Fortmeier I Hoeller T Hornung T. Assessment of tumor thickness in melanocytic skin lesions: comparison of optical coherence tomography, 20-MHz ultrasound and histopathology. Dermatol (Basel Switzerland) (2011) 223(2):161–8. doi: 10.1159/000332845 22024981

[14] Music M Hertl K Kadivec M Pavlović M Hocevar M. Pre-operative ultrasound with a 12-15 MHz linear probe reliably differentiates between melanoma thicker and thinner than 1 mm. J Eur Acad Dermatol Venereol JEADV (2010) 24(9):1105–8. doi: 10.1111/j.1468-3083.2010.03587.x 20236207

[15] Lassau N Lamuraglia M Koscielny S Spatz A Roche A Leclere J. Prognostic value of angiogenesis evaluated with high-frequency and colour Doppler sonography for preoperative assessment of primary cutaneous melanomas: correlation with recurrence after a 5 year follow-up period. Cancer Imaging Off Publ Int Cancer Imaging Society (2006) 6:24–9. doi: 10.1102/1470-7330.2006.0009 PMC1693780 16644502

[16] Yu N Wu L Su J Huang K Liu S Lu L. Preoperative ultrasound-guided incisional biopsy enhances the pathological accuracy of incisional biopsy of cutaneous melanoma: A prospective clinical trial in chinese patients. J Ultrasound Med (2022) 41(11):2841–8. doi: 10.1002/jum.15972 35233820

[17] Chaput L Laurent E Pare A Sallot A Mourtada Y Ossant F. One-step surgical removal of cutaneous melanoma with surgical margins based on preoperative ultrasound measurement of the thickness of the melanoma. Eur J Dermatol EJD (2018) 28(2):202–8. doi: 10.1684/ejd.2018.3298 29620001

[18] Polańska A Jenerowicz D Paszyńska E Żaba R Adamski Z Dańczak-Pazdrowska A. High-frequency ultrasonography-possibilities and perspectives of the use of 20 MHz in teledermatology. Front Med (2021) 8:619965. doi: 10.3389/fmed.2021.619965 PMC7937737 33693015

