# Corrigendum: The utility of high-frequency 18 MHz ultrasonography for preoperative evaluation of acral melanoma thickness in Chinese patients

**DOI:** 10.3389/fonc.2024.1331419

**Published:** 2024-03-28

**Authors:** Nianzhou Yu, Kai Huang, Yixin Li, Zixi Jiang, Siliang Liu, Yuancheng Liu, Xiaowan Liu, Zeyu Chen, Renliang He, Tianhong Wei

**Affiliations:** ^1^ Department of Dermatology, Hunan Engineering Research Center of Skin Health and Disease, Hunan Key Laboratory of Skin Cancer and Psoriasis, Xiangya Hospital, Central South University, Changsha, Hunan, China; ^2^ National Clinical Research Center for Geriatric Disorders, Xiangya Hospital, Central South University, Changsha, Hunan, China; ^3^ State Key Laboratory of High Performance Complex Manufacturing, College of Mechanical and Electrical Engineering, Central South University, Changsha, China; ^4^ Department of Dermatologic Surgery, Dermatology Hospital of Southern Medical University, Guangzhou, Guangdong, China; ^5^ Department of Ultrasound, Xiangya Hospital, Central South University, Changsha, Hunan, China

**Keywords:** acral melanoma, high-frequency, ultrasonography, thickness, preoperative

Incorrect Ethics statement

In the brief research report article, there was an error in the Ethics statement section.

A correction has been made to the Ethics statement section and now states:

“The studies involving humans were approved by the Ethics Committee of Xiangya Hospital of Central South University. The studies were conducted in accordance with the local legislation and institutional requirements. Written informed consent for participation in this study was provided by the participants’ legal guardians/next of kin.”

The authors apologize for this error and state that this does not change the scientific conclusions of the article in any way. The original article has been updated.

