# Supplementary material for: The utility of high-frequency 18 MHz ultrasonography for preoperative evaluation of acral melanoma thickness in Chinese patients
Source: Front Oncol. 2023 Oct 5;13:1185389. doi: 10.3389/fonc.2023.1185389 (PMC10585136; doi:10.3389/fonc.2023.1185389)
Supplement: Supplementary file 1 [file DataSheet_1.pdf]

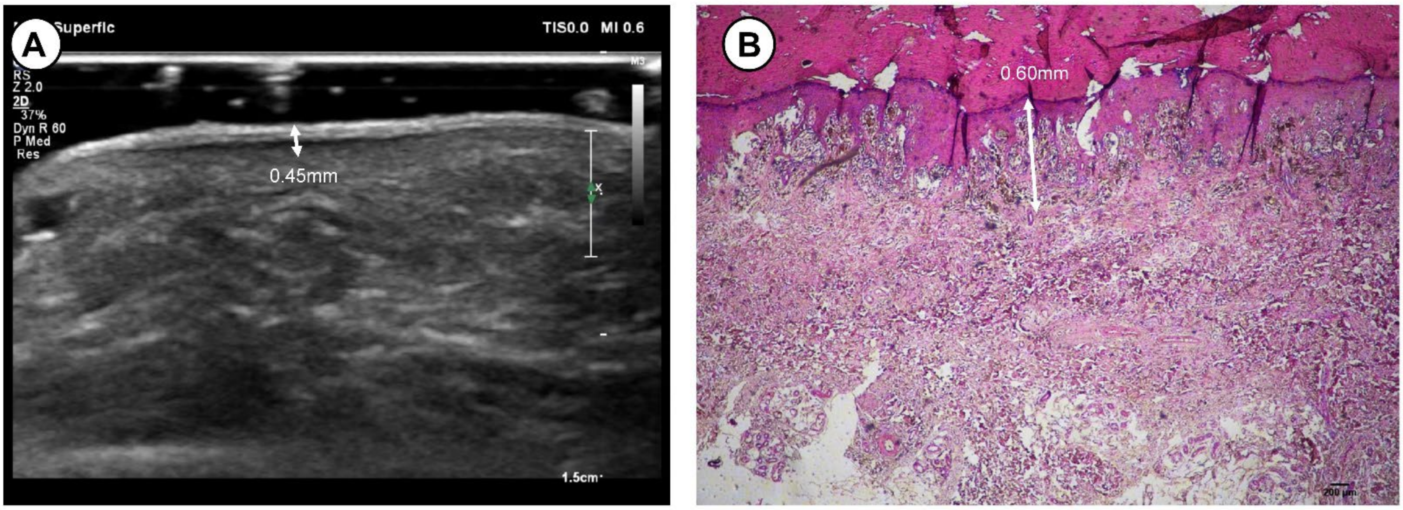

Figure S1. One Superficial spreading melanoma located on the plantar side: (A) ultrasound pictures and (B) pathological pictures.
